# Validation of a method for the dry preservation and rehydration of *Anopheles gambiae* sensu lato for parity analysis to assess the impact of vector control measures in the field

**DOI:** 10.1186/s13071-023-05866-2

**Published:** 2023-07-15

**Authors:** Elizabeth Pretorius, Mojca Kristan, John Bradley, Eunice Teixeira da Silva, Harry Hutchins, Fatucha Barri, Ansumane Cassama, Sainey Ceesay, Mamadou Ousmane Ndiath, Amabelia Rodrigues, James G. Logan, Anna Last, Robert T. Jones

**Affiliations:** 1 Department for Disease Control, London School of Hygiene and Tropical Medicine, London, UK; 2 Clinical Research Department, London School of Hygiene and Tropical Medicine, London, UK; 3 Projecto de Saúde Bandim, Bissau, Guinea-Bissau; 4 Ministério de Saúde Pública, Bissau, Guinea-Bissau; 5 Medical Research Council Unit, London School of Hygiene and Tropical Medicine, Fajara, The Gambia; 6 Arctech Innovation, Dagenham, London, UK

**Keywords:** Age-grading, Malaria, Parity, Vector control, Mosquito, *Anopheles*, Guinea-Bissau

## Abstract

**Background:**

As the control of malaria remains heavily dependent on vector management interventions, it is important to understand the impact of these on mosquito populations. Age-grading is a valuable tool for this; however, logistical challenges in remote, resource-poor areas make current methodologies difficult to incorporate into clinical trials and routine surveillance. Our aim was to validate a methodology that could be easily implemented in such settings. Using dried mosquito specimens instead of freshly killed ones, we validated the commonly used ovarian tracheation technique for assessing population age structure.

**Methods:**

Laboratory-reared *Anopheles coluzzii* mosquitoes with known parity status were dry preserved in silica gel for up to 12 weeks and rehydrated prior to parity assessment. The results were compared to parity results for freshly killed mosquitoes from the same colony. Preserved, field-caught *Anopheles gambiae* sensu lato (s.l.) from Guinea-Bissau were assessed by three different assessors blinded to each other’s scores. An overall index of agreement was calculated using inter-rater reliability of all assessor pairings. The impact of preservation time was investigated using a one-way ANOVA to look for differences in assessor agreement over three time periods.

**Results:**

The parity status was correctly identified for 90% of dry preserved and rehydrated insectary-reared *An. coluzzii* and for 98% of freshly killed insectary-reared *An. coluzzii*. The inter-rater reliability was highest (0.94) for freshly killed *An. coluzzii*. The results for all time points showed excellent strength of agreement between assessors. For field-caught *An. gambiae* s.l., the overall index of agreement between all three assessors was 0.86 (95% confidence interval 0.78–0.93), indicating almost perfect agreement. There was no significant difference between assessor agreement between time frames.

**Conclusions:**

Dry preserving and rehydrating *Anopheles* mosquitoes provides an alternative to using freshly killed mosquitoes to assess the efficacy of a control intervention in remote settings where it is logistically difficult to dissect fresh specimens. This method also provides the flexibility required for parity assessment to be done on larger scales over bigger areas.

**Graphical Abstract:**

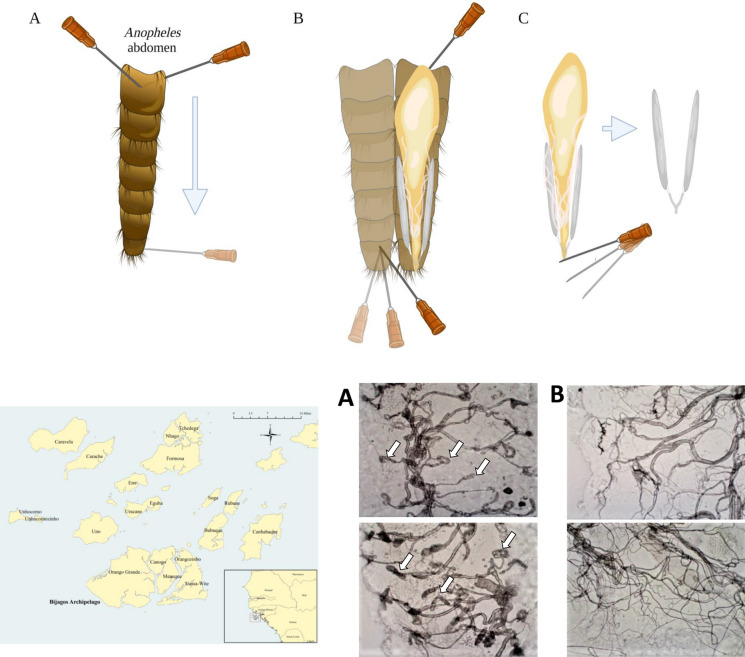

## Background

Despite the half-century of successful campaigns and control interventions against malaria that have been implemented during the elimination era, there are still approximately 250 million cases of malaria and 600,000 deaths due to the disease annually, most of which are seen in children under the age of 5 years [1]. Control efforts remain largely dependent on vector control strategies, mainly through the widespread distribution of insecticide-treated nets and indoor residual spraying. However, whilst great strides have been made, progress has stalled in recent years [2–4]. As well as continued efficacy monitoring of existing measures, novel interventions for the control of mosquito populations are required to maintain the progress made thus far and for progress to continue.

The overall aim of current vector control measures is to reduce the number of potentially infectious bites by targeting mosquitoes of blood-feeding age. This decreases both the likelihood of the extrinsic incubation period (the period between the parasite being taken up in the blood meal and developing to its infective sporozoite stage) being completed and the mosquito’s ability to complete gonotrophic cycles, leading to fewer mosquitoes in the next generation [5]. The parity rate of a mosquito population is a key indicator in studies assessing the entomological impact of interventions [6]. It represents the average age of the mosquito population, assuming that the population is stable, i.e. recruitment and loss are similar [7]. Mosquitoes that have taken a blood meal and laid eggs are termed parous mosquitoes, whereas those that have not laid eggs are termed nulliparous mosquitoes. If an intervention is successful in killing a proportion of mosquitoes of blood-feeding age, then the proportion of parous mosquitoes within that population will decrease [6]. Small reductions in parity can lead to large decreases in malaria transmission [8].

There are various techniques for the assessment of mosquito age. Techniques such as chromatographic analysis of cuticular hydrocarbons, transcriptomic profiling, and mid-infrared and near-infrared spectroscopy show promise. However, they are often expensive and logistically challenging to use in remote, resource-poor settings [6, 9–12]. Morphological assessment of mosquito ovaries has been most frequently used in vector control studies. There are multiple techniques for the morphological classification of the parity of a mosquito. The Polovodova ovarian separation technique and ovarian oil injection can be used to estimate the number of gonotrophic cycles completed by a mosquito [10, 11]. However, these techniques are technically difficult and thus require a high level of skill and expertise. The technique most frequently used, the ovarian tracheation technique, was first described in 1962 [13]. It is the most technically and logistically simple of the morphological methods, generating a binary parous/nulliparous outcome. It only requires a stereomicroscope, a compound microscope and dissection tools.

Whilst the ovarian tracheation technique is relatively simple to perform, there are challenges to its use in certain settings. The technique requires mosquitoes to be freshly killed prior to dissection. Mosquitoes that have died 1 or more days before dissection are either too brittle to dissect or too decomposed to assess. Thus, all of the specimens trapped overnight in a field study need to be dissected the same day. If a study involves trapping at multiple and distant sites at the same time, as might be required for an intervention trial with multiple clusters, dissection can only be achieved with multiple parity assessment teams. This has cost implications as it requires trained personnel, available equipment, and a high level of quality control; it also decentralises oversight. By dry preserving mosquito samples soon after they have been collected and rehydrating them later in a central laboratory, greater oversight and quality control of the procedure can be achieved in a study, in addition to reduced transport and equipment costs.

Preservation methods have been explored using laboratory-reared mosquitoes for dissection [14]. These include the dissection of mosquitoes dried in silica gel; preserved in fixatives including formalin, ethanol, Bouin’s and Carnoy’s solutions; or frozen. Preserved mosquitoes were rehydrated prior to dissection, whilst frozen specimens were dissected after thawing without rehydration [14]. All three methods were feasible, but little information was given on the effect of the length of preservation time on the accuracy of the parity scoring, and none was provided on the accuracy of the parity scores for field-caught mosquitoes. In the present study, we use insectary-reared mosquitoes, in addition to field-caught mosquitoes collected in the Bijagós Archipelago, Guinea-Bissau, to validate the dry preservation and rehydration method for parity analysis. We also investigate whether the length of time that mosquitoes are preserved for affects the accuracy of parity assessment.

## Methods

Validation of the desiccation and rehydration method for parity assessment was carried out in two stages: with female *Anopheles coluzzii* N’gousso strain reared at the London School of Hygiene and Tropical Medicine (LSHTM), and with field-caught female *Anopheles gambiae* sensu lato collected on the Bijagós. *Anopheles coluzzii* N’gousso strain is a laboratory strain that was established from mosquitoes collected in the field around Yaoundé, Cameroon, in 2006 [15]. Confirmatory polymerase chain reaction (PCR) was done at LSHTM to verify the species [16].

### Validation using laboratory-reared *An. coluzzii* mosquitoes

#### Mosquitoes

*Anopheles coluzzii* were maintained under a 12:12-h light:dark cycle at 27 ± 2 °C and 70 ± 10% relative humidity at the insectaries at LSHTM. The mosquitoes were provided with a constant supply of 10% glucose.

#### Validation design

To evaluate the methodology, mosquitoes with known parity status were prepared. Cages contained either parous or nulliparous mosquitoes. Parous mosquitoes were generated by blood-feeding females aged 3–5 days. The female mosquitoes were provided a blood meal on 2 consecutive days, then allowed to lay eggs once. Females that did not take a blood meal were removed from the cages. Nulliparous female mosquitoes, which were of the same age, 3–5 days, were not provided a blood meal. When the mosquitoes were approximately 8–10 days old (after egg-laying in the parous cages), they were killed using ethyl acetate and dry preserved in 15-ml universal tubes containing silica gel beads and cotton wool.

To investigate whether the preservation period affected the accuracy of the parity assessment, mosquitoes were dry preserved for 1, 2, 6, 9 and 12 weeks. The temperature at the time of preservation was 30 °C and the relative humidity 62%. A subset of parous and nulliparous mosquitoes from all of the cages were removed, killed, and immediately dissected, using the ovarian tracheation technique, for comparison with those that had been dry preserved and rehydrated [13]. Over 100 mosquitoes were used at a 1:1 ratio for freshly killed and dry preserved mosquitoes at each time point.

#### Rehydration and dissection of dry preserved mosquitoes

**Fig. 1:**
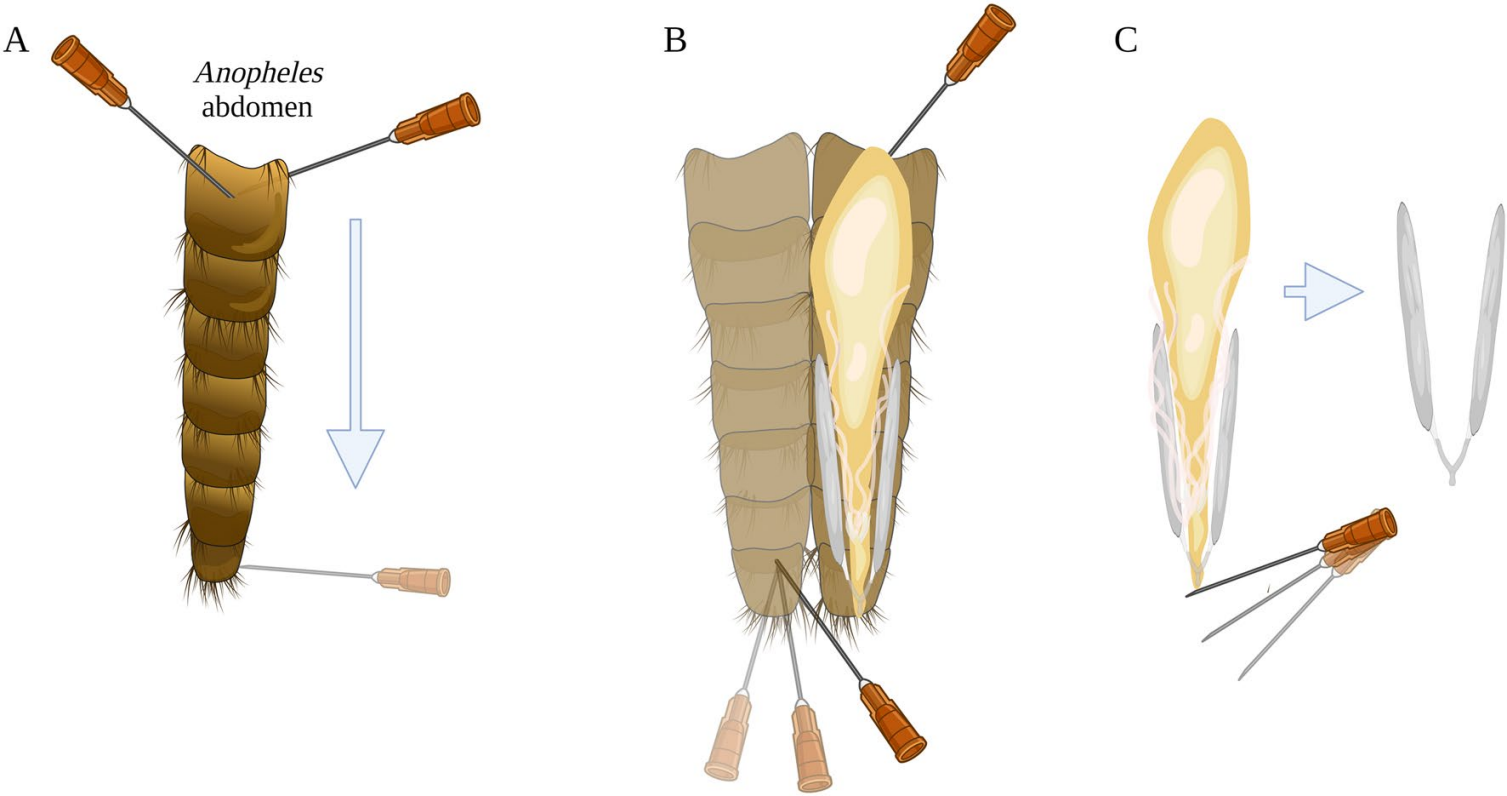
**a**–**c** Dissection of ovaries from dried and rehydrated mosquitoes after removal of head and thorax with 28-gauge needles. **a** Lateral abdominal incision; **b** peeling back the cuticle to reveal the midgut, Malpighian tubules and reproductive organs; **c** identification and isolation of the ovaries

**Fig. 2:**
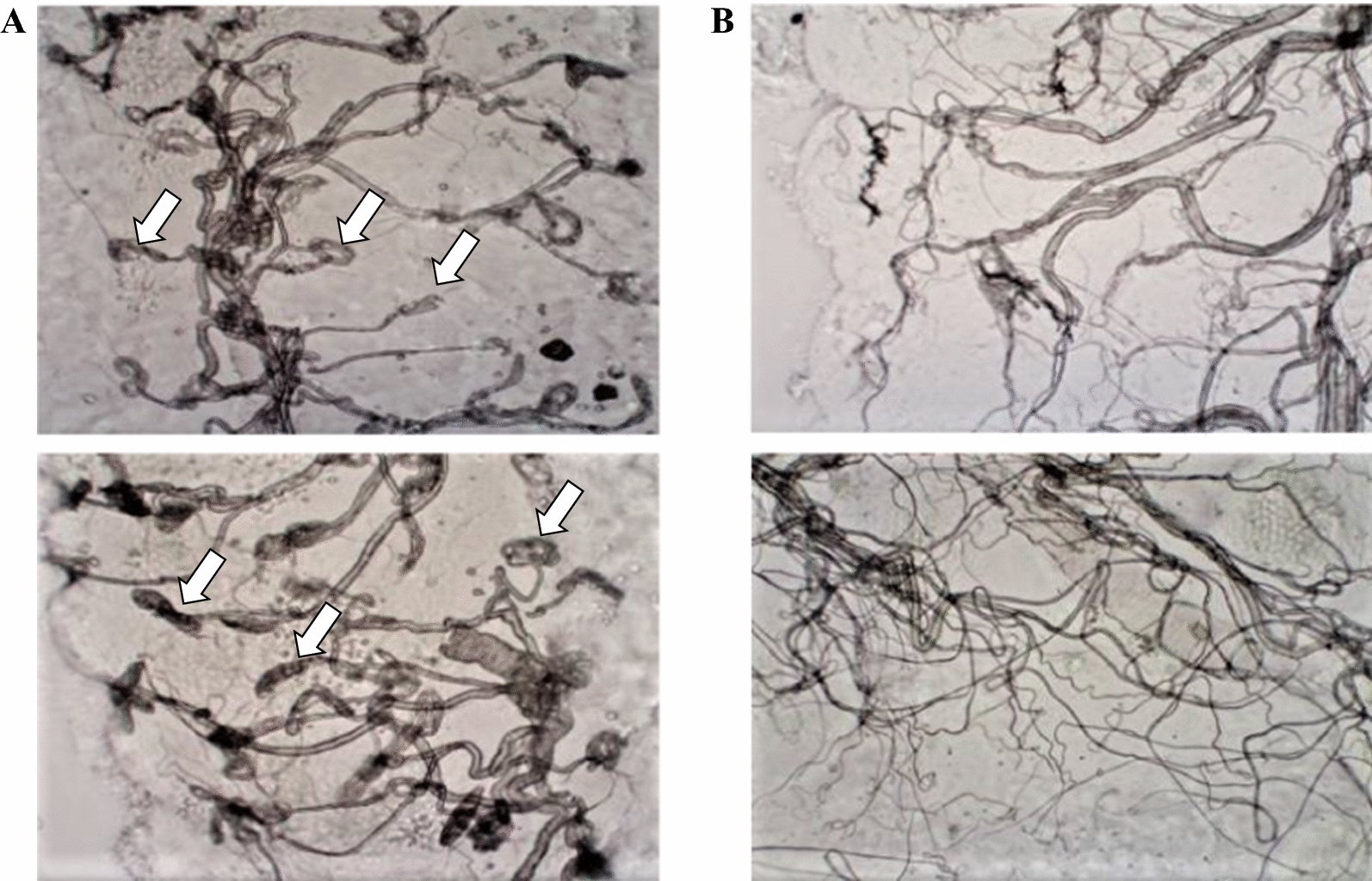
Dried and rehydrated ovaries of *Anopheles coluzzii* showing tracheation. **a** Tracheation in a nulliparous ovary showing skeins (arrows); **b** tracheation in a parous ovary showing unravelled tracheoles. (Photographs taken by EP)

Prior to rehydration, mosquitoes were randomly assigned to individual 1.5-ml microcentrifuge tubes by a third party. The assessors were blind to parity status until after the assessment had been completed. Mosquitoes were rehydrated by soaking in 1 ml of 20% liquid detergent solution (Multipurpose detergent; Teepol Products, Orpington, Kent, UK) for 20 min. They were then transferred to distilled water for a further 20 min. The mosquitoes were rehydrated in batches of 40. For mosquito dissection, a specimen was placed onto a microscope slide and a drop of distilled water applied. Firstly, using two 28-gauge needles, the head and thorax were removed. A lateral incision was then made along the length of the abdomen (Fig. 1). The abdomen was opened to expose the internal organs. The ovaries were identified and carefully isolated. The ovaries were transferred to a clean drop of distilled water and allowed to dry in the same way as in the ovarian tracheation method performed on freshly killed mosquitoes. Once dry, the ovaries were examined under a compound microscope at ×40 magnification to determine the presence or absence of skeins (Fig. 2).

All of the dissections were performed by one individual. The parity status of each pair of ovaries was then evaluated by two assessors. Once both assessors had determined the parity status of all of the mosquitoes for each time point, the true parity status was revealed. Mosquitoes that could not be dissected due to them being too brittle, or because of a technical error during dissection, were classified as ‘loss to dissection’. Mosquitoes that could not be dissected because they had begun to decompose were classified as ‘loss to decomposition’.

### Validation using field-caught *An. gambiae* s.l. mosquitoes

*Anopheles* mosquitoes were caught in the Bijagós as part of ongoing studies undertaken by our group [17, 18]. The mosquitoes were caught by using indoor CDC Miniature Light Traps (model 512; John W. Hock, Gainesville, FL) and previously described methodology [19]. All of the *An. gambiae* s.l. caught were identified using previously described morphological keys [20], killed using acetone, and stored dry using the same method as described above. The temperature at the time of preservation was 29 ± 3 °C and the relative humidity 80 ± 11%. Mosquitoes were then transported to a central laboratory where 350 randomly selected mosquitoes from across the archipelago were rehydrated and dissected. The ovaries were then scored by the first assessor. Photographs of the ovaries were taken using a microscope camera (Brunel Eyecam Plus; Brunel Microscopes, Chippenham, Wiltshire, UK) and then sent to two additional assessors for independent scoring by each. All of the assessors were blind to each other’s scores.

Confirmatory PCR–restriction fragment length polymorphism was performed on a subset of 45 samples at the Medical Research Council Unit The Gambia at LSHTM [16]. DNA was extracted by using a QIAcube extraction robot (QIAcube; QIAgen, Venlo, the Netherlands) in accordance with the manufacturer’s protocol.

### Statistical analysis

For insectary-reared mosquitoes, the results of each assessor for freshly killed mosquitoes were compared to those for dry preserved and rehydrated mosquitoes at each time point using a continuity-adjusted chi-square test. The inter-rater reliability (IRR), which indicates the extent to which two assessors agree, was calculated using Cohen’s kappa statistic [21]. For strength of agreement, Landis and Koch [22] proposed the following interpretation of the kappa coefficient (κ): ≤ 0 = poor, 0.01–0.20 = slight, 0.21–0.04 = fair, 0.41–0.60 = moderate, 0.61–0.80 = substantial, and 0.81–1 = almost perfect.

For field-caught mosquitoes from Guinea-Bissau, κ was calculated for all assessor pairs. An overall index of agreement was then calculated using the arithmetic mean of all-pair κ. To assess the impact of period of desiccation (i.e. the dry preservation period between sacrifice and rehydration of mosquitoes) on assessor agreement, the results were split into three time frames: 16–70 days, 71–90 days, and 91–110 days. An overall index of agreement was calculated using the method described above for each time frame. A one-way ANOVA was then carried out to investigate the difference between the index of agreement calculated for each time frame. All of the statistical analyses were performed using SAS 9.4 (SAS Institute, Cary, NC).

## Results

### Validation using insectary-reared *An. coluzzii* mosquitoes

The process of desiccating and rehydrating mosquitoes can result in some damage or decomposition that prevents them from being scored for parity status. The highest loss to dissection or decomposition, 17.4% (22 individuals), was after 1 week of preservation, prior to rehydration (Table 1). By comparison, only 1.4% (two individuals) of freshly killed mosquitoes could not be scored due to loss to dissection.

**Table 1:** Loss to dissection or decomposition for lab-reared mosquitoes assessed at London School of Hygiene and Tropical Medicine (LSHTM)

	Time preserved (weeks)	Total randomised	Total successfully dissected and assessed	Loss to dissection (%)	Loss to decomposition (%)
Freshly killed	–	138	136	2 (1.4)	0 (0.0)
Dry preserved and rehydrated	1	132	110	5 (4.5)	17 (12.9)
	2	133	128	2 (1.5)	3 (2.3)
	6	110	107	3 (2.7)	0 (0.0)
	9	116	115	1 (0.9)	0 (0.0)
	12	113	101	10 (8.8)	2 (1.8)

Following unblinding of the parity status of the insectary-reared mosquitoes, the IRR was calculated to assess the level of agreement between the assessors’ scores. The accuracy of scoring mosquitoes that were freshly killed and those that had been dry preserved was also compared for all time points up to 12 weeks. Overall, the proportion of freshly killed mosquitoes that were correctly scored was 0.98 (0.98 parous and 0.97 nulliparous) when averaged over the two assessors. The proportion that was correctly scored after dry preservation and rehydration was lower, at 0.90 (0.90 parous and 0.90 nulliparous).

Whilst the ability to accurately determine parity status was reduced in the desiccated samples, there was no clear trend of reduced accuracy as time of preservation increased. For assessor 1, there were significant differences in the accuracy of parity scoring between mosquitoes that were freshly killed and those that were dry preserved and rehydrated at three time points: 1 week, 2 weeks and 12 weeks (Table 2). For assessor 2, there was a significant difference between these mosquitoes at the 6-week time point. The IRR was highest, at 0.94, for freshly killed mosquitoes, indicating that the assessors were more likely to give the same score for these mosquitoes.

**Table 2:** Results of the validation of the methodology carried out using insectary-reared *Anopheles coluzzii* mosquitoes at LSHTM

	Time preserved (weeks)	Assessor 1						Assessor 2						IRR^b^
		Correctly identified as parous			Correctly identified as nulliparous			Correctly identified as parous			Correctly identified as nulliparous			
		*n/N* (prop.)	(95% CI)	Continuity- adjusted χ^2^^a^	*n/N* (prop.)	(95% CI)	Continuity- adjusted χ^2^^a^	*n/N* (prop.)	(95% CI)	Continuity-adjusted χ^2a^	*n/N* (prop.)	(95% CI)	Continuity- adjusted χ^2^^a^	
Freshly killed	–	65/66 (0.98)	(0.95–1.00)	-	68/70 (0.97)	(0.93–1.00)	-	65/66 (0.98)	(0.92–1.00)	-	68/70 (0.97)	(0.93–1.00)	-	0.94
Dry preserved and rehydrated	1	43/54 (0.80)	(0.69–0.90)	χ^2^ = 9.7, *P* = 0.002*	44/45 (0.98)	(0.93–1.00)	χ^2^ = 0.0*,* *P* = 1.000	60/65 (0.92)	(0.86–0.99)	χ^2^ = 1.6*,* *P* = 0.203	43/45 (0.96)	(0.89–1.00)	χ^2^ = 0.0*,* *P* = 1.000	0.83
	2	48/58 (0.83)	(0.73–0.92)	χ^2^ = 7.6*,* *P* = 0.006*	48/51 (0.94)	(0.88–1.00)	χ^2^ = 0.1*,* *P* = 0.716	70/76 (0.92)	(0.86–0.98)	χ^2^ = 1.8*,* *P* = 0.173	45/52 (0.87)	(0.77–0.96)	χ^2^ = 3.5*,* *P* = 0.062	0.76
	6	53/59 (0.90)	(0.82–0.97)	χ^2^ = 2.9*,* *P* = 0.087	42/49 (0.86)	(0.76–0.95)	χ^2^ = 3.6*,* *P* = 0.058	55/58 (0.95)	(0.89–1.00)	χ^2^ = 0.4*,* *P* = 0.522	40/49 (0.82)	(0.71–0.92)	χ^2^ = 6.5*,* *P* = 0.011*	0.79
	9	60/67 (0.90)	(0.82–0.97)	χ^2^ = 3.2*,* *P* = 0.072	46/48 (0.96)	(0.90–1.00)	χ^2^ = 0.9*,* *P* = 0.362	62/66 (0.94)	(0.88–1.00)	χ^2^ = 0.8*,* *P* = 0.362	41/46 (0.89)	(0.80–0.98)	χ^2^ = 3.2*,* *P* = 0.072	0.87
	12	49/53 (0.92)	(0.82–0.98)	χ^2^ = 0.4*,* *P* = 0.242	38/48 (0.79)	(0.65–0.89)	χ^2^ = 8.2*,* *P* = 0.004*	50/52 (0.96)	(0.91–1.00)	χ^2^ = 0.04*,* *P* = 0.833	45/48 (0.94)	(0.87–1.00)	χ^2^ = 1.2*,* *P* = 0.665	0.77

*CI* Confidence interval,* prop*. proportion

* Significant difference between correctly identified parity status of freshly killed and preserved mosquitoes at that time point

^a^*df* = 1 for all continuity-adjusted chi-square tests

^b^Inter-rater reliability (*IRR*) calculated using Cohen’s kappa statistic [21]

### Validation using field-caught *An. gambiae* s.l. mosquitoes

Field-caught mosquitoes were scored for parity by three assessors. Out of the 357 mosquitoes analysed, 324 were scored by all three assessors. The scores were categorised according to three time frames (Table 3). Since it was not possible to know the true parity status of the field-caught specimens, the ability to score them correctly was determined using the degree of agreement between the assessors. The index of agreement between the three assessors, who were blind to each other’s scores, was 0.83 (95% CIs 0.74–0.92), indicating almost perfect agreement between them. There was no significant difference between the index of agreement between the three time frames [*F*_(2,6)_ = 0.15, *p* = 0.866].

**Table 3:** Total number of mosquitoes dissected and identified by all three assessors. Index of agreement calculated by using the arithmetic mean of all-pair kappa statistics

Period of desiccation (days)	Total dissected	Total assessed by all three assessors	Total unable to be identified by one or more assessors (%)	Index of agreement (95% CIs)
16–70	69	63	6 (9.5)	0.82 (0.78–0.87)
71–90	141	127	14 (9.9)	0.84 (0.79–0.89)
91–110	140	134	6 (4.3)	0.82 (0.55–1.00)

*Anopheles gambiae* sensu stricto (s.s.) (4.4%), *Anopheles gambiae* s.s./*Anopheles coluzzii* hybrids (11.1%) and *Anopheles melas* (84.4%) were present within the subset of 45 samples identified to species level using PCR-restriction fragment length polymorphism (Table 4).

**Table 4:** Number of species within the *Anopheles gambiae* complex identified using polymerase chain reaction–restriction fragment length polymorphism

*Anopheles* species	*n* (%)
*Anopheles gambiae* sensu stricto (s.s.)	2 (4.4)
*Anopheles gambiae* s.s./*Anopheles coluzzii* hybrid	5 (11.1)
*Anopheles melas*	38 (84.4)

## Discussion

This study demonstrates that the ovarian tracheation method can be performed on dry preserved and rehydrated mosquitoes, and can be used in a remote setting with little infrastructure [6]. It builds on previous work that validated the methodology in both a laboratory and field setting [14]. Laboratory-reared *An. coluzzii* mosquitoes could be successfully rehydrated and scored after being dry preserved for up to 12 weeks. Similarly, field-caught *An. gambiae* s.l. from Guinea-Bissau, which were dry preserved in the field for up to 16 weeks, were successfully rehydrated and scored. This technique would be beneficial in remote, resource-poor settings, like the Bijagós, where transport is challenging. By centralising the parity analysis, greater oversight and quality control can be achieved in clinical trials or during routine surveillance.

The 20% soap solution that was used during the rehydration step disrupts the phospholipid layer of cells, enabling water to permeate them [23]. Once this has taken place, specimens may be vulnerable to decomposition or disintegration [14]. Decomposition due to this is most evident in the mosquito digestive tract, with the ovaries seemingly more resistant. The high loss to decomposition in the laboratory-reared mosquitoes rehydrated at the 1 week time point (12.9%) may have been due to poor mosquito handling during randomisation or slow speed of dissection. Mosquitoes were rehydrated in batches of 40. Therefore, at the 1-week time point, some of the mosquitoes may have been in distilled water for a number of hours prior to their dissection. This delay was reduced as the dissection skills of the individual dissecting the mosquitoes improved. As freshly killed mosquitoes are not rehydrated, the risk of decomposition is low. It is challenging to estimate the maximum time that mosquitoes can undergo rehydration prior to decomposition commencing, as the conditions at the time of preservation and rehydration will play a role in this. When the temperature and humidity are higher, faster decomposition is expected; therefore, it may be necessary to rehydrate smaller batches of mosquitoes under these conditions. As it is also crucial to ensure that silica gel continues to properly preserve specimens, the use of self-indicating silica gel is recommended. The use of self-indicating silica gel will also help to protect specimens from decomposition should there be a handling error during the preserving step, for instance, if a sample tube is not properly capped or there is too much moisture in a tube before samples are added to it.

Compared to freshly killed mosquitoes, the abdominal tissues of rehydrated specimens lack elasticity, which makes it harder to dissect them. The rehydrated laboratory-reared mosquitoes showed a 0.9–8.8% loss to dissection, whereas the freshly killed mosquitoes showed a 1.4% loss. The decrease in the loss to dissection at the 2-, 6- and 9-week time points indicated that the dissection technique improved with time. Loss to dissection at the 12-week time point can be attributed to specimens becoming more brittle after being preserved for longer. Field-caught mosquitoes showed a similar loss to dissection for the 16- to 70-day and 71- to 90-day time frames (9.5% and 9.9%, respectively); however, the percentage loss was halved to 4.28% for the 91- to 110-day time frame. This may have been due to the improved skill of the individual carrying out the dissections, or more favourable conditions when the specimens were preserved or rehydrated. If this methodology is to be used in a study, the loss to dissection should be taken into consideration. When planning a study, the experience of the individual(s) performing the dissection should be taken into consideration; if they are highly skilled, a 10–20% loss to dissection should be factored in.

With regards to the mosquitoes reared in the insectary, those that were incorrectly scored by assessor 1 did not always correspond to those that were incorrectly scored by assessor 2. For instance, at the 2-week time point, assessor 1 correctly assessed significantly fewer lab-reared than freshly killed mosquitoes. This difference was not seen in the results of assessor 2 at the same point. The divergence in the IRR at this time point also illustrates this, indicating that while one assessor may incorrectly identify the parity status of a mosquito, the other assessor is likely to correctly identify the parity status of the same mosquito. For field-caught mosquitoes, the index of agreement was relatively stable, and indicated almost perfect agreement throughout. However, the wider 95% CI for the 91- to 110-day time frame indicated less agreement between assessors. When designing a study in which it is planned to dry preserve specimens, a cut-off point for the maximum length of time specimens can be preserved prior to rehydration should be determined. The conditions in which specimens are dry preserved and rehydrated will vary depending on the setting, so undertaking trials to assess the methodology prior to large-scale implementation is important.

As described above, the tissues of rehydrated mosquitoes lack the elasticity of freshly killed ones; these specimens are more fragile, which makes their dissection more challenging. The ovaries often stick to the cuticle, which makes it difficult to see them. To ensure the individual undertaking the dissection is able to successfully identify the ovaries and gently remove them, thorough training and practice is required. It is recommended that there should be multiple trained assessors to ensure that the results are of the highest quality, and, wherever feasible, a third-party assessor should arbitrate in scoring specimens for which parity assessments differ.

The technique of dry preserving and rehydrating mosquitoes could be used for other established methodologies. Ungureanu [14] undertook further dissections, successfully removing the salivary glands and identifying sporozoites in infective mosquitoes, and performed more complex morphological assessments of mosquito ovaries, such as Polovodova’s ovariole separation technique. However, little information on these methods was provided, except that the dissections were mainly performed on recently dried specimens. The ovarian tracheation method gives a binary outcome, i.e. parous or nulliparous, and does not give information on the number of gonotrophic cycles completed by a mosquito. Validation of the ovariole separation technique for rehydrated mosquitoes is required. Future work is also needed to investigate whether it is possible to perform other complex methods of morphological identification, such as ovarian oil injection, on dry preserved and rehydrated specimens [10, 11].

Unlike the laboratory-reared mosquitoes, we could not be certain of the parity status of the field-caught *An. gambiae* s.l., which left us heavily reliant on assessor IRR. Ideally, an assay using mosquitoes of known parity status at the study site should precede validation using field-caught mosquitoes. However, in the Bijagós it was not possible to carry out any assessment of laboratory-reared mosquitoes due to the lack of an available colony. In addition, further validation is needed using vector species other than those within the *An. gambiae* complex, the focus of the present study. A further limitation of this study is that all of the dissections were made by one individual, which raises concerns with respect to generalisability. However, since completing the validation work, multiple individuals have been successfully trained and are now able to remove mosquito ovaries using the method described here.

## Conclusions

*Anopheles gambiae* s.l. mosquitoes can be dry preserved for up to 110 days and successfully rehydrated to allow parity assessment. Wherever possible, freshly killed mosquitoes should still be used as a first option. However, dry preservation and rehydration may be used in remote settings where parity assessment of freshly killed mosquitoes is not feasible. This method may also be a good alternative for large-scale concurrent surveillance. In such circumstances, this technique enables greater quality control and oversight of data collection.

## Data Availability

All the data presented in this manuscript are available from the corresponding author upon reasonable request.

## Abbreviations

IRR: Inter-rater reliability
LSHTM: London School of Hygiene and Tropical Medicine
